# RESEARCH ISSUES AND INITIATIVES: NIEHS Funds Human BPA Research

**DOI:** 10.1289/ehp.117-a541

**Published:** 2009-12

**Authors:** Angela Spivey

**Affiliations:** **Angela Spivey** writes from North Carolina about science, medicine, and higher education. She has written for *EHP* since 2001 and is a member of the National Association of Science Writers

With $14 million in stimulus funds from the American Recovery and Reinvestment Act, the NIEHS is bolstering a coordinated effort to produce data on bisphenol A (BPA) that will help refine our understanding of whether the general population’s current exposures to the chemical pose a health risk. Used in producing plastics, BPA can leach into food and beverages from everyday items such as food storage containers, water bottles, and baby bottles.

The NIEHS has used the stimulus monies to fund 10 two-year studies on the potential contribution of low-dose BPA to problems such as obesity, diabetes, reproductive disorders, asthma, sexually dimorphic behaviors, cardiovascular diseases, and prostate, breast, and uterine cancer. Those grants augment ongoing work on BPA by researchers in the NIEHS Intramural Division and at the National Toxicology Program (NTP).

Many animal studies suggest exposure to low doses of BPA during critical periods of fetal development may result in adverse reproductive, behavioral, and carcinogenic changes over the long term. However, fewer studies have examined whether or how the effects seen in animals translate to humans. In 2008 the U.S. Food and Drug Administration (FDA) declared BPA safe, with an updated ruling pending at press time. But regardless of what the FDA decides at this point, scientists involved in BPA research agree more human data are needed for the compound.

“Policy makers and regulatory agencies such as the FDA are constantly looking at new data, and we’re hoping that the data we will provide in the next two years will have a significant impact in helping them [continue to assess] the health effects of this chemical,” says Jerry Heindel, a health scientist administrator at the NIEHS.

Coordinating the new effort with research already under way will yield a more comprehensive understanding of BPA while also maximizing resources, says Linda S. Birnbaum, director of the NIEHS and the NTP. “We saw the stimulus package award as a real opportunity to bring together the ongoing NIEHS work, the NTP work, and these new projects to clearly answer the question of how much of a problem BPA may or may not be,” Birnbaum says. In total, including the stimulus funds, the institute will invest approximately $30 million over two years on BPA-related research.

Many of the awardees met with institute scientists involved in ongoing BPA research in October 2009. “Having the key players talking to one another as they begin new research efforts will stimulate collaboration, create opportunities to share resources, and encourage researchers to develop reliable and reproducible methods that will allow for a comprehensive assessment of the human health effects of BPA,” Heindel explained in an NIEHS press release.

The group will continue to meet periodically and share data and tissue samples. For example, one of the new grantees, B. Paige Lawrence, an associate professor at the University of Rochester School of Medicine, is studying whether BPA influences immune-mediated diseases, but some of the data from her study may also provide clues to the chemical’s potential role in cancers. “The same types of cells and pathways that fight viral infections also detect and destroy tumor cells,” she says.

The new effort is crucial to getting results from human studies quickly, says grantee Kim Harley, an epidemiologist at the University of California, Berkeley, who will study BPA levels and health outcomes in a birth cohort of 300 children followed through age 12. “We haven’t focused on BPA before,” she says, “but we have this valuable cohort as well as urine samples stored, so with this grant we can measure BPA levels and start to see the effects in children all the way to puberty.”

